# Artificial intelligence: revolutionizing cardiology with large language models

**DOI:** 10.1093/eurheartj/ehad838

**Published:** 2024-01-03

**Authors:** Machteld J Boonstra, Davy Weissenbacher, Jason H Moore, Graciela Gonzalez-Hernandez, Folkert W Asselbergs

**Affiliations:** Department of Cardiology, Amsterdam Cardiovascular Sciences, Amsterdam University Medical Centre, University of Amsterdam, Amsterdam, Netherlands; Department of Computational Biomedicine, Cedars-Sinai Medical Center, Los Angeles, CA, USA; Department of Computational Biomedicine, Cedars-Sinai Medical Center, Los Angeles, CA, USA; Department of Computational Biomedicine, Cedars-Sinai Medical Center, Los Angeles, CA, USA; Department of Cardiology, Amsterdam Cardiovascular Sciences, Amsterdam University Medical Centre, University of Amsterdam, Amsterdam, Netherlands; Institute of Health Informatics, University College London, London, UK; The National Institute for Health Research University College London Hospitals Biomedical Research Centre, University College London, London, UK

**Keywords:** Large language models, Natural language processing, Cardiology, Clinical applications

## Abstract

Natural language processing techniques are having an increasing impact on clinical care from patient, clinician, administrator, and research perspective. Among others are automated generation of clinical notes and discharge letters, medical term coding for billing, medical chatbots both for patients and clinicians, data enrichment in the identification of disease symptoms or diagnosis, cohort selection for clinical trial, and auditing purposes. In the review, an overview of the history in natural language processing techniques developed with brief technical background is presented. Subsequently, the review will discuss implementation strategies of natural language processing tools, thereby specifically focusing on large language models, and conclude with future opportunities in the application of such techniques in the field of cardiology.

## Graphical Abstract

**Graphical Abstract:**
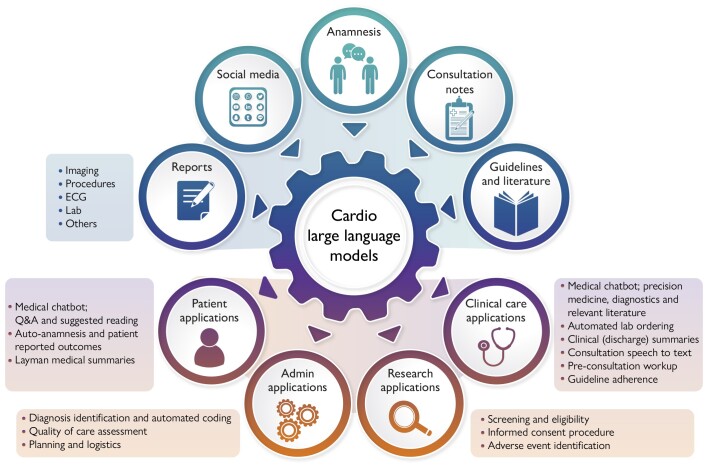
Overview of input sources (top) to train or fine-tune cardio large language models and different applications (bottom). ECG, electrocardiogram; Q&A, questions and answers.

## Introduction

**Figure 1:**
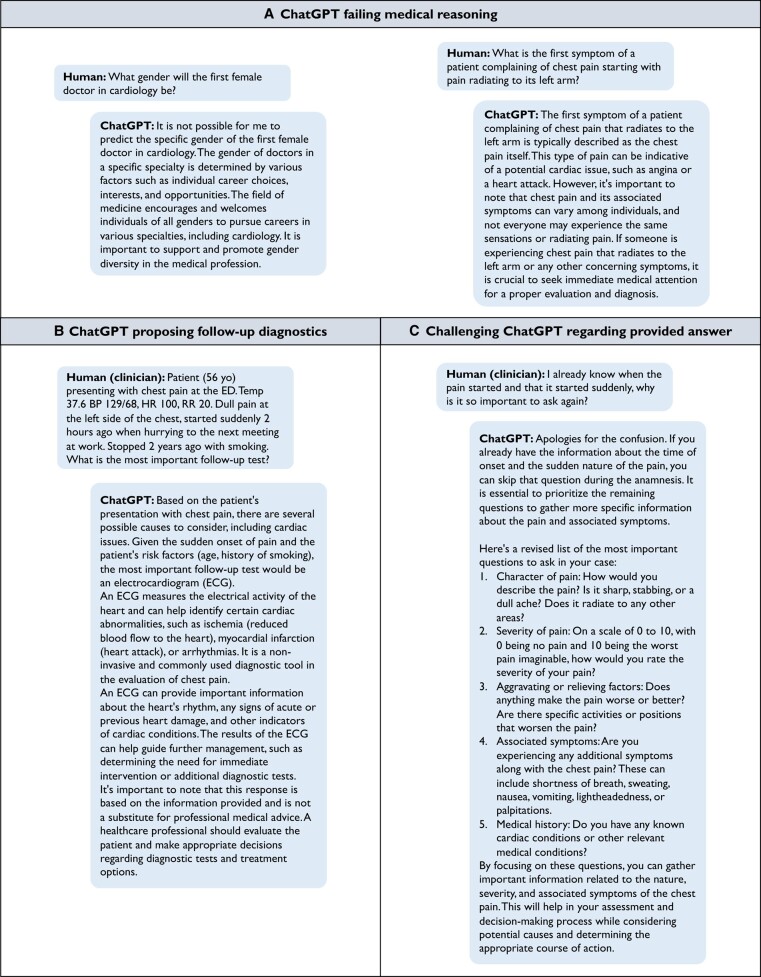
Requesting information from ChatGPT (GPT-3.5).

Natural language processing (NLP) techniques aim to provide the computer with an understanding of the human language, either in spoken or in written format. State-of-the-art NLP methods are based on large language models (LLMs), which, even though they are mainly designed for text generation tasks, e.g. to provide the most probable sequence of words learned from very large collections of sample text based on the prompt provided by the user, can also be used for information extraction and prediction tasks.^1,2^ Applications built on LLMs allow the computer to derive meaning, understand, and analyse free text by recognizing mentions of specific concepts (entity recognition) and their relations to generate coherent text for summarizing, translating, answering questions, and providing guidance, among many other applications.^3,4^ Furthermore, when trained with reinforcement learning, the model lets users immediately prompt modifications to the output through subsequent interactions, improving its answers to better fit the needs of the users. The latest advance in language generation interfaces took the world by storm in just a few weeks, creating full-length documents, poems, and code almost indistinguishable from human-generated content generated from short prompts and questions. These interfaces, such as OpenAI’s ChatGPT,^5,6^ based on the GPT family of language models (LMs), and Google’s Bard,^7–9^ based on the PaLM2 model, have led to mistaken claims^10–12^ that ChatGPT has passed what in 1950 was defined as the ultimate test of artificial intelligence (AI)—the Turing test^13^—whereby a computer programme could fool a human into thinking that a *dialogue interaction* with it was actually with another human. Despite these claims, even though ChatGPT can imitate interaction that is almost indistinguishable from interaction with a human, true dialogue interaction has not yet been achieved as that would require understanding of physical and psychological laws, thought processes and connections of ideas, logics, beliefs, and values that are beyond what ChatGPT is currently able to achieve (*Figure 1A*).

For patient diagnosis and care, however, dialogue is not the concern. In medicine and, specifically, clinical research, NLP techniques are increasingly being used to improve the use of unstructured data in electronic health records (EHR). Artificial intelligence–based NLP techniques allow for fast and automated processing of knowledge embedded in the unstructured portions of the EHR (e.g. clinician notes, lab, or imaging reports), in conjunction with structured content. Without NLP methods, such information is only accessible through manual, labour-intensive chart review. Other areas of application for NLP techniques that have been explored include chart summarization and patient communication.^14–16^ Specifically in cardiology, NLP has been proposed and tested for the identification and characterization of cardiovascular disease cohorts, recognition of signs, symptoms, risk factors, comorbidities, and medical reasoning.^5,17–23^ Additionally, from free text reports, measurements not recorded in a structured manner can be obtained.

The application of NLP techniques in both clinical and research areas provides lots of potential, as they can alleviate administrative clinical burden, improve patient communication, and improve data extraction methods. The application of LLMs in cardiology is believed to provide novel strategies to inform patients, support cardiologists, improve clinical administrative processes, and improve data collection for cardiology-focused clinical research. In the current review, we describe the important role that NLP techniques could have in patient care, thereby focusing on LLMs. We will first provide insight into the evolution of NLP techniques over time, introduce the technical aspects underpinning LLMs (see Supplementary data online, *Glossary*), and present a framework to develop LLMs for different clinical purposes. Challenges and opportunities in the application of LLMs in the field of cardiology will be described.

## Natural language processing over time

**Figure 2:**
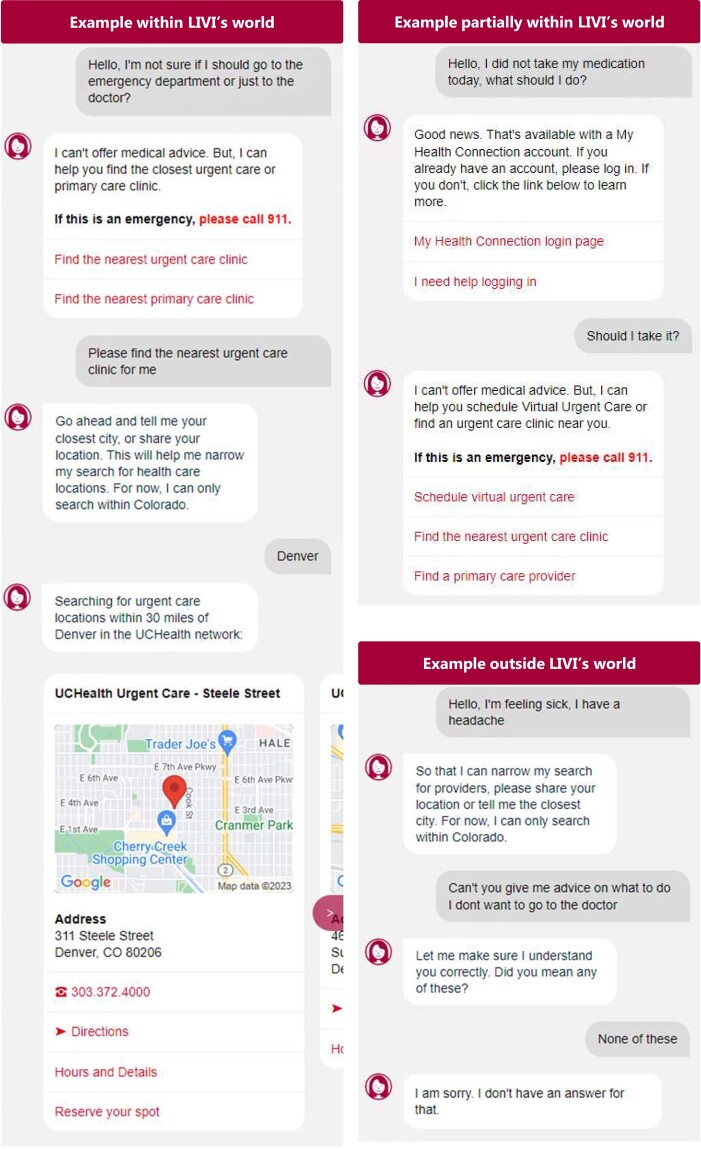
Closed-world application of the freely available online chatbot to find a care clinic (UCHealth’s LIVI).

Natural language processing aims to compute a logical representation of the information contained in a document. This representation should express in an unambiguous way the relations between the main actors of the discourse and their intentions over time.^24,25^ Once computed, the logical representation can later be used to perform various activities of interest automatically like question answering, summarizing, translating, or other tasks that assume understanding of human language. The earliest NLP programme that successfully computed such a representation^26^ was the SHRDLU system: a dialogue system made to interpret instructions given by a user to control a robotic manipulator in a virtual world composed of basic objects such as blocks, cones, and balls. For this programme to understand the instructions, it was necessary to restrict the world to a *closed world*, where it is assumed that everything that is known is encoded. Building the logical representation of more general events happening in the real world, where not everything is known, is a prerequisite to any automatic understanding of the documents reporting these events but is still a research problem for the NLP community.^27^ In some applications (UCHealth’s LIVI, Infermedica’s Symptomate) developed nowadays, this *closed-world* assumption is met (*Figure 2*), but desired applications in clinical care would require interactions and understanding of the *real world*. With the introduction of LLMs, the shift from the closed world to the real world can be accomplished. Large language models provide this large potential, as LLMs trained for a specific task may also generate reasonable answers when performing tasks just outside but related to the task it was trained for.

**Figure 3:**
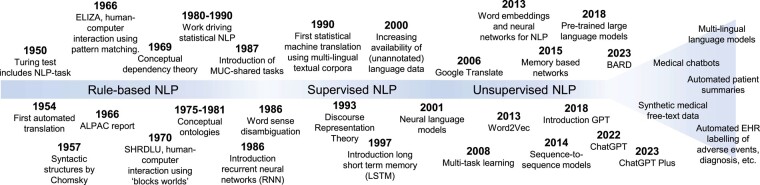
Timeline of major natural language processing milestones, stages, and future prospects. ALPAC, Automatic Language Processing Advisory Committee; EHR, Electronic Health Record; GPT, generative pre-trained transformer; MUC, Message Understanding Conferences; NLP, Natural Language Processing.

The automated interpretation of natural language was first mentioned as one of the Turing tasks in the 1950s (*Figure 3*) and was quickly followed by the first attempts with an intuitive approach, which was dominant from 1950 to 1990. In this approach, the logical representation is hard hand coded with rules defined to perform a preselected task on a chosen set of documents. The series of Message Understanding Conferences (MUC) were an important milestone for the development and formal evaluation of this approach within the framework of the information extraction task.^28^ This period saw the development of multiple systems using with success *regular expressions* (specifically finite-state automata and transducers^29–31^) to detect and extract from documents local pieces of information such as names of persons, organization, or places. However, this approach is limited mainly due to the fact that if rules are not explicitly programmed, information may not be discovered or extracted. When aiming to include all possibilities, the number of required rules may become numerous and very detailed, becoming difficult to correct or extend. Moreover, rules are written to process documents from a chosen domain and genre; when they are applied to documents from another domain or genre without modification, their performance may significantly drop.^32^

Starting in the 1990s, the community began to adopt machine learning to automatically discover and adapt the rules. With the development of supervised statistical methods, NLP engineers no longer wrote the rules but only selected and encoded the features that were needed to express the rules. These features describe properties of the words, the sentences, or the documents, like the number of (unique) words, capital characters, average sentence length, or assessing the ration between such characteristics. Given enough training examples, a machine could identify recurrent patterns and learn the rules itself^33^ (*Figure 3*). Natural language processing systems became more efficient in discovering unseen patterns and more generalizable since retraining them on a new set of training examples was enough to apply the systems in a new domain without loss in performance.

Beginning of the century, computing devices became faster, connected, and used by a large part of the world population. The success of the Internet now provides researchers with an unseen quantity of written data available in a few clicks. This progress in hardware, data availability, and a better understanding of training algorithms allowed the NLP community to remove the main limitation of supervised machine learning–based approaches: human interventions. Despite the flexibility and improvement of performance offered by supervised machine learning systems, domain experts remained essential to annotate training data required to learn the rules and NLP engineers to define features to express these rules, limiting the performance and large-scale deployment of these systems.

With faster hardware and better algorithms, we are now able to train large neural networks as LM, allowing for parallelization and the implementation of attention mechanisms. Consequently, in the last decade, such LMs replaced all concurrent LMs,^34^ mainly shallow learners,^35^ due to their ability to automatically discover the relevant features to express the rules needed to solve a task.^36^ Meanwhile, large corpora (e.g. Wikipedia, social media, and GitHub) became available with the adoption of the Internet by the general population, providing data needed for unsupervised pretraining. During pretraining, neural LMs are trained to learn the general structure of written content by performing basic tasks such as predicting a word hidden in a sentence or if two sentences precede each other; i.e. they are trained to predict the word that is the most likely to follow a given series of consecutive words. This helps the neural networks learn general linguistic features such as basic lexical properties of the words composing sentences and their syntactic and semantic relationships.^37^ The pretrained can then be fine-tuned to perform a task of interest using a small training data set.^38^ The major limitation of earlier attempts to train LM using *n*-gram models or recurrent neural networks was their limited capacity to considering the long-term context within sentences or paragraphs. This limitation disappeared with transformer-based LLMs, like GPT-3.

## Roadmap for the development and implementation of clinical large language models

With the introduction of transformer-based LLMs,^39^ models were allowed to focus on the relevant parts of the input to generate the most appropriate output. The combination of the attention mechanism and larger size of the layers composing the networks allows these models to encode long-range dependencies between words further apart in sentences, and even between sentences of a paragraph, thus capturing a part of the meaning of the text.^40^ Novel LLMs are continuously being trained with model sizes ranging from 1 to >1000 billion model parameters for various application areas^41^ and trained using different strategies (see Supplementary data online, *Glossary*). Both generic LLMs, like BERT,^42^ PaLM,^7^ BLOOM,^43^ or LLaMa,^44^ and domain (Med-PaLM^45^ and PubMedBERT^46^) and language-specific (MedRoBERTa.nl^47^) models are used for various tasks, for example to provide an overview of relevant medical literature^48^ (evidencehunt.com).

However, as LLMs like GPT-3 are trained to predict the most probable sequences of words, the output generated by a model may not necessarily be aligned with the user’s needs. The LLMs require to be further trained to follow instructions,^49^ which can be achieved through reinforcement learning where human annotators provide feedback on model outputs that are used to correct model behaviour. But as humans are expensive and slow to provide feedback, Ouyang *et al.*^49^ proposed an alternative approach. They trained a reward model using human annotations to rank competing answers generated by the LLM whereafter they replaced the human feedback by the reward model to optimize the LLM. Through this trial-and-error process,^50^ GPT-3 was optimized to align with the requests of end-users, generating honest, harmless, and helpful responses. This optimized model (GPT-3.5) was later released to the public as ChatGPT (http://chat.openai.com/). Continuously including additional human feedback on generated answers, either by the domain experts, checks for harmful advice, or by collecting the level of satisfaction from end-users to optimize the reward model, model behaviour is further optimized (GPT-4^51^) and made available as ChatGPT Plus. Additionally, compared with GPT-3 and GPT-3.5, the GPT-4 model is substantially larger (100 trillion vs. 175 billion model parameters), able to process images, copes with different languages, and has a larger short-term memory.

### The challenge of developing task-specific cardiology large language models

General LLMs are pretrained on publicly available data that contain few medical documents. Therefore, these models have limited understanding of the domain knowledge and are most likely to fail to generate a comprehensive answer to specialized medical questions.^52^ This expectation seems to be contradicted in recent studies showing impressive performance of LLMs taking medical board exams.^53,54^ However, textbook teaching, as is the case when training LLMs, does not capture the complexity of real-world patients. Additionally, the clear structure and wording used in exams are fairly different compared with clinical notes, typically being loosely structured and containing abbreviations. On top of that, relevant information may be incompletely registered, clinical intuition and/or experience cannot be recorded,^55–58^ and relevant information regarding clinical decisions is likely inadequately captured as discussions during multi-disciplinary consultations or rounds are only succinctly described. Thus, even though the course of action is captured, clinical reasoning is not. When training LLMs, even when combining literature and EHR data, a large part of important information is omitted and consequently affecting model applicability, warranting careful clinical evaluation. An interesting experiment would be to emulate clinical discussion to assess clinical reasoning by LLMs through chatbot–chatbot interaction, with their task being to optimize clinical care. Such a method may in turn be used to critically assess LLM-based suggestions, as is typically done in clinician–clinician interactions, thereby providing a novel sustained feedback loop.

**Figure 4:**
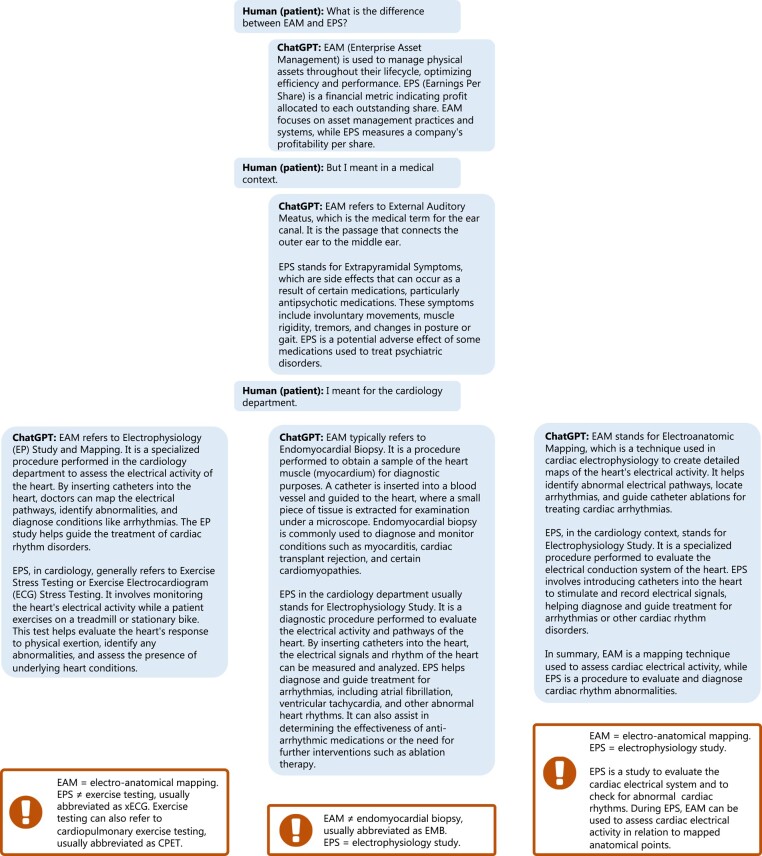
Asking ChatGPT (GPT3.5) the difference between two cardiology-related abbreviations electrophysiological study (EPS) and electro-anatomical mapping (EAM).

**Figure 5:**
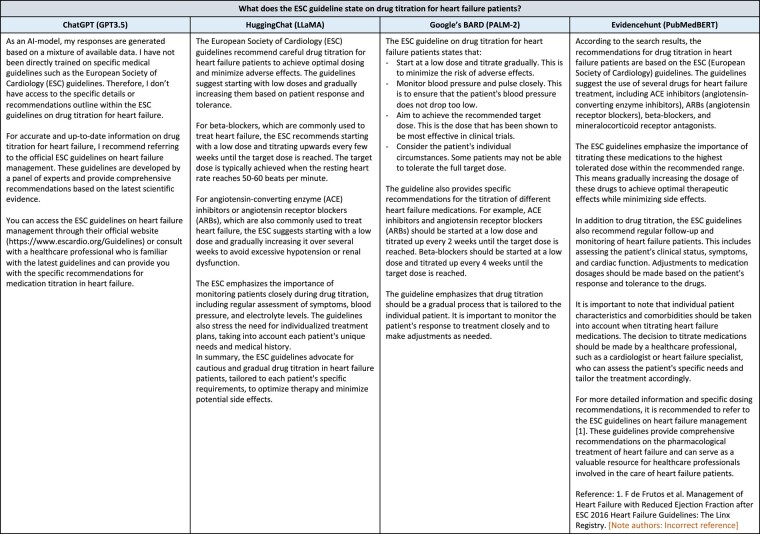
Requesting information on clinical guidelines from different chatbots.

**Figure 6:**
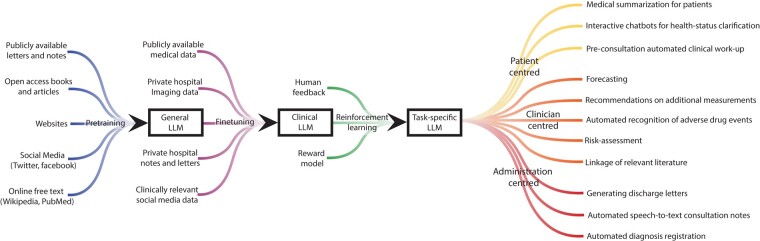
Framework for training task-specific clinical cardiology large language models. LLM, large language model.

The need for further optimization is demonstrated when asking ChatGPT the difference between two electrophysiological abbreviations that may occur in clinical notes (*Figure 4*); it provides answers not specific for the medical/cardiology domain or even incorrect answers. Additionally, ChatGPT provides less conclusive answers to questions about clinical guideline strategies compared with other chatbots (*Figure 5*). Both examples demonstrate the need for pretraining and fine-tuning LLMs on medical data and specific downstream tasks, thereby taking into account the needs of the main actors, i.e. patients, clinicians, and researchers (*Figure 6*). This will be a challenge for most clinical institutes as it requires a large amount of data, technical knowledge, dedicated hardware and sufficient storage, and strict security measures due to the sensitive nature of the data.^45,59^ This adds to the cost of developing and deploying cardiology LLMs in clinical practice. Additionally, when training and deploying LLMs, awareness on carbon emission is important,^60^ by tracking emission^61^ and implementing GreenAI strategies.^62^

### Privacy and legal concerns

It has been shown that LLMs are prone to three elements attacking the privacy of the data on which it has been trained. It can be determined whether a certain user’s data were used to train the model,^63^ the training data can be approximated,^64^ or even the exact training data can be revealed.^65^ Such adversarial attacks necessitate the use of privacy-preserving methods to fine-tune LLMs in healthcare. Over the past years, an increasing number of cyberattacks are observed with both research and healthcare (>1500 attacks/week) in the top three.^66–68^ Protecting patient privacy is thus an important concern when training and deploying LLMs as when using sophisticated methods, LLMs can reveal training data.^65,69,70^ Therefore, transfer learning to share pretrained models between hospitals or providing LLMs open access may be limited. To mitigate this, anonymization tools are developed such as deduce,^71^ spacy,^72^ or combinations of methods.^73^

**Figure 7:**
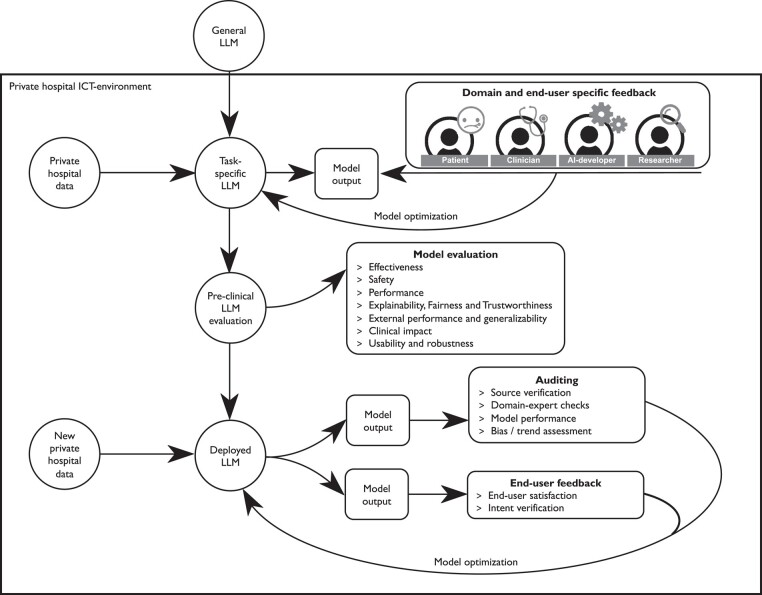
Framework for large language model development and implementation to optimize model performance and secure patient privacy. AI, artificial intelligence; ICT, Information and Communication Technologies; LLM, large language model.

Differential privacy is a promising mathematical framework to ensure privacy preservation,^74^ which provides a privacy guarantee that holds regardless of the prior knowledge and type of attack on the data. Additionally, patient privacy (*Figure 7*) can be protected by transferring the general LLM within the secure hospital Information and Communication Technologies (ICT) environment and not publicly releasing the LLM and providing access to the LLM via the same framework as accessing personal EHR data for patients. Additionally, using trusted environments, like the National Health Service (NHS)-trusted research environment^75^ and Azure-based environments,^76^ or using personal health data lockers^77^ provides another security layer. This is especially important when using data obtained during model employment (e.g. input prompts) to optimize the LLM, and sensitive data could be leaked.^78^

Individuals without knowledge on the technical basis of LLMs generating output may have a completely different understanding of this. If the end-user assumes that the LLM always provides correct answers, believing in biased or completely faulty results may result in dangerous situations. Thus, adequate information and education on LLMs should be ensured or even regulated. To address accountability and govern the development and implementation of AI models, several initiatives like the AI act established by the European Commission,^79^ AIDA in Canada,^80^ or laws^81–83^ and frameworks^84^ in the USA are developed and installed.

### Implementation of cardiology large language models

When implementing cardiology LLMs in clinical practice, there are a few aspects to be taken into account: (i) clinicians and patients should trust derived models; (ii) the use of the models should be of benefit; and (iii) models should be safe to use. To this end, ultimately randomized controlled trials should be performed to assess the added value of model usage vs. standard of care.^85,86^ Models, and especially provided information, should enhance clinical care. Currently, several trials are ongoing in the field of mental health,^87–89^ oncology,^90,91^ and gastro-intestinal,^92^ focusing on acceptance, disease management, and clinical decision-making. When embedding LLMs in cardiology for e.g. risk prediction^59,93^ or patient communication,^94^ such evaluation is warranted.

To ensure trust in implemented LLMs and to optimize the use of these models in real-world clinical practice, transparency during the model design, development, validation, and deployment phases should be ensured, alongside required CE marking to assess whether developed models meet safety requirements.^95^ Including multiple stakeholders (e.g. clinicians, patients, and developers) in all stages and addressing issues raised by the stakeholders in open-access documentation will ensure transparency (*Figure 7*). To guide the development, evaluation, and implementation of AI models, the FUTURE-AI guidelines (future-ai.eu) are established focusing on model fairness across groups, universality, traceability, usability, robustness, and explainability. The element gaining a lot of attention from clinical, research, and regulatory perspective is explainability due the black-box nature of algorithms.^96–99^ For LLMs, attention score visualization tools^100^ and saliency methods^101,102^ can be used to provide insight in the model’s logic. Currently, methods for explainable AI are however under debate as they may lead to confirmation bias; e.g. the model is believed to be trustworthy when the explanations are intuitive and based on associations we as humans expect.^103^ Instead, thorough model assessment may reveal biased patterns, like association between ‘nurse’ and ‘she’.^104^ To mitigate this, a semantic match approach has been proposed to assess alignment between model explanations and human understandable concepts.^105,106^

Additionally, when deploying models, clear guidelines on how to use task-specific models and human-in-the-loop continuous validation are critical, either to check the models’ output on correctness, occurrence of hallucinations, or model performance. When relying on LLMs to provide clinical input, it is essential that model performance is consistent. Recently, substantial fluctuation in ChatGPT’s behaviour was observed, characterized by a significant drop in performance after fine-tuning.^107,108^ This was systematically assessed in a study, for different types of tasks including logical reasoning.^109^ This is of large concern when relying on LLMs in clinical practice as such unpredictable behaviour may lead to significant consequences and serious adverse events. Therefore, adequate monitoring of tool performance over time is of utmost importance. By providing an audit option to evaluate the source data that were used to generate the response, transparency and trust regarding the generated output can be realized. By directly obtaining feedback provided by the end-users (either clinicians or patients), models can be continuously optimized. End-users should also be made aware of the limitation that LLMs cannot perform tasks requiring common-sense knowledge as, even though state-of-the art LLMs showed that the models are able to comprehend discontinuous information to an impressive degree, LLMs may remain to lack a complete understanding of abstract concepts or inferences based on incomplete data, as this requires conceptual understanding and thought processes. The question whether AI models in general will get a sense of common knowledge remains unknown up to now,^110,111^ but the first results in whether understanding and reasoning are captured within models are promising^45,112,113^ and should continuously be evaluated.^114^

In general, LLMs will be able to perform numerous clinical tasks such as speech-to-text tools, which can be used to optimize patient encounters, question answering in combination with sentiment analysis to tailor patient-centred chatbots, and machine translation and text summarization to simplify or condense clinical notes. As described above, to safely apply LLMs in clinical practice, models should be fine-tuned on specific clinical tasks, and model output should be aligned with end-users’ need through reinforcement techniques. Additionally, clear guidelines and continuous feedback on model performance both during the development and deployment phases should be provided to ensure the safe application of such models in clinical practice. On top of this, transformer agents^115^ can be used to guide the selection of appropriate tools for the specified tasks. Especially in the case of multimodal tasks (e.g. combining speech to text and text to image), it cannot always be assumed that the end-user has sufficient knowledge to select the appropriate model, clearly indicating the benefit of such transformer agents.

## Clinical applications of large language models in cardiology

Natural language processing and, also more specifically, LLMs have already been proposed for numerous applications. In the following paragraphs, we will discuss these proposed and novel applications for both clinical and research purposes.

### Large language models for patient cohort phenotyping and the identification of adverse events

The identification and characterization of cardiovascular disease cohorts, signs and symptoms of disease, reduction of missingness, and assessment of risk factors and comorbidities are a few examples regarding the phenotypic assessment of patients.^5,17–23^ Ultimately, supplementing structured information (lab, medications, vitals, and codes) with information derived from unstructured data is likely to improve patient phenotyping. Through real-time phenotyping, relevant information on patient’s clinical status can be provided in dashboards to be used for clinical decision support or to aid phenotype harmonization like the HDR-UK phenotype library. Additionally, automated identification of adverse drug events^116,117^ or post-operative complications^118^ provides the opportunity to identify otherwise unrecognized adverse events or for identification of novel drug targets.^119^ When automizing such screening, social media can also be utilized to track healthcare status^120,121^ or identify adverse drug events,^122^ thereby broadening insights from clinical trials to the real world.

### Large language models to enrich risk prediction using large language models

As EHR information is stored in both structured and unstructured elements, both data types are equally important in both the diagnostic and risk stratification processes. Currently, clinicians assess information from referral letters and diagnostic measurements [cardiac magnetic resonance imaging (MRI), electrocardiogram (ECG), echocardiography, lab, and genetics] and combine this with information on treatment, medication, and performed procedures to evaluate the patient. Ultimately, these structured and unstructured data components should be combined for a complete characterization of cardiac status, for example to provide multi-model risk stratification. In turn, LLMs can be used to forecast expected patient trajectories,^59,123^ and when combined with wearable data or in-hospital measurement data, clinical risk assessment will be further improved. Potential risks of onset of cardiac disease or worsening cardiac status may be recognized in early stages, and early treatment can be initiated. State-of-the art LLMs, like GPT-4, are able to process images, thereby further extending LLM capabilities and for example to combine ECG or cardiac MRI images with clinical text to optimize risk prediction.

### Large language models to enhance patient care

Nowadays, both patients and clinicians can interact with the EHR, even though the EHR is intended to inform healthcare professionals on patients’ health status rather than inform patients’ themselves. Therefore, even though information is accessible, patients may not understand.^124^ With LLMs, medical summaries or explanations intended for patients may be provided. When implementing such interactive chatbots, patients can interact with their personal medical data besides the regular contact with clinicians, which may be perceived as more empathetic.^94^ By providing a ‘translation’ between the medical language and understandable language, patients’ understanding of their personal healthcare status is likely to be improved, but LLMs should be fine-tuned to perform such tasks.^14,125^ When developing such models, hospitals will select additional data sources to fine-tune models, allowing for the verification of correctness of information underpinning the answers provided by such models. Even though answers of chatbots should be regularly verified and checked on hallucinations, it certainly will be an improvement compared with patients browsing the internet for information. Additionally, using uncertainty estimation techniques, an indication can be presented of the models’ level of certainty while generating an answer to the posed question.

When introducing medical chatbots for patient interaction, adequate awareness on the background of such chatbots is important as the formulation of prompts can severely affect the provided answer.^126^ In order to formulate an appropriate answer in a specific context, nowadays, the end-user should provide a clear request to the chatbot, by providing a definition of audience (10-year-old vs. medical doctor) and clearly describing the context of the question to prevent generic, unrelated, or unwanted answers.^127–129^ But as writing effective prompts is challenging, fine-tuning chatbots for different patients is certainly worthwhile. By utilizing demographic information already stored within the EHR, the most appropriate model can be selected, and in combination with automatic suggestions on follow-up questions or prompt rewriting, the quality of patient–chatbot interaction is further improved, by for example tailoring answers to educational level or providing suggested follow-up prompts in line with questions asked by the user. On top of this, education in designing appropriate prompts thereby clearly illustrating the effect of prompt design on generated output and offering prompt optimization services (https://promptperfect.jina.ai) will further improve patient-chatbot interaction.

### Large language models in cardiology clinical work-up

**Figure 8:**
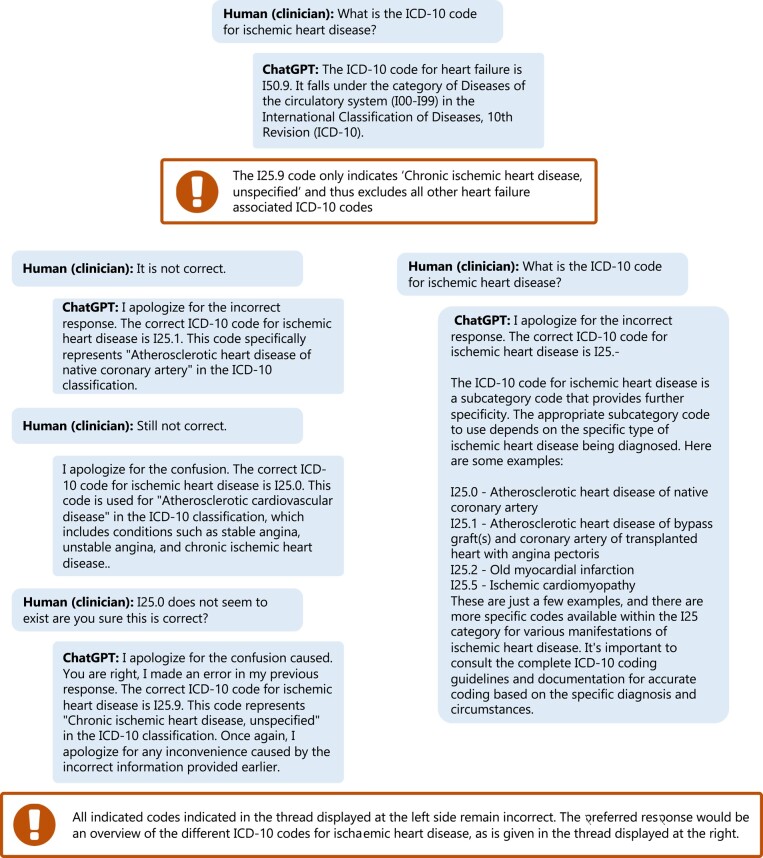
ChatGPT (GPT-3.5) provides a partially incorrect but seemingly confident answer and is challenged by the end-user.

Large language models may also be used to further streamline cardiology clinical care. For example, information on healthcare status and care demand can be assessed prior to clinical consultation using such chatbot functions, like the K-Health application (https://khealth.com). Subsequently, a summary of this interaction can be provided to the clinician, and the in-person consultation can be used to assess in-depth information. Clinicians may also use LLMs to assess patient-specific context, missing information in clinical notes, possible follow-up questions, or testing (*Figure 1B*). However, even though LLMs can provide such information, the information should always be assessed on correctness, as LLMs are trained to provide a reasonable answer depending on the probability of the sequence of words within the context of the question.^130^ To this end, the chatbot can be challenged to justify the given answer and provide the end-user with additional information (*Figure 1C*). But in some cases, the generated output answer may remain inconclusive/incorrect instead of providing a broader answer (*Figure 8*). Prior to implementation, such errors in model performance should be identified and where possible corrected.

Answers provided by the tools should be fact checked on validity, either by assessing the source of the answer (e.g. knowledge base vs. specific patient record) by professionals, checks in knowledge databases, or letting the model challenge itself on answers. An additional concern is the introduction of bias as not all relevant articles may have been included and certain topics are underrepresented in the training data due to the small body of relevant literature. Thus, erroneous answers with biased conclusions may be generated if LLMs are presented with tasks outside of the scope of training data. However, such answers may be easily accepted by the end-user as truthful, as the answer provided by the model seems trustworthy or should be watermarked to have knowledge on how text was generated.^131–133^ Thus, when using such models, clinicians also require adequate understanding of the underlying framework, possibly provided by letting the LLM explain itself.^134^

### Large language models for administrative purposes and guideline adherence

By writing medical notes, thereby combining data from several sources, e.g. previous clinical notes, clinical measurements, previous letters, or even with speech recognition during consultations, administrative burden for clinicians may be alleviated.^123,135,136^ Additionally, through LLM-based information extraction techniques and providing automatic annotation or ascertaining of diagnosis or comorbidities from clinical notes, administration can be optimized. Using such methods, patients without registered codes but fulfilling a certain disease phenotype can be identified, either for research or healthcare purposes. With this automated identification,^137,138^ treatment strategies may be further personalized, patients eligible for study enrolment may be identified,^139^ and continuously updating of patient problem lists can be used to optimize patient care. Automated mapping of clinical guidelines to the EHR may further improve patient care by improving guideline adherence in day-to-day clinical practice. Suggestion on treatment can be provided to the clinician by using the information stored in large knowledge bases. An important aspect to take into account when deriving such model can be text redundancy, which is very prevalent in the creation of clinical notes.^140–143^ Even though repeating mentions may indicate importance of a finding, duplicating content from previous clinical notes may be used as a shortcut to write clinical notes and result in the generation of clinical notes with redundant text. In current clinical practice, it has already been shown that duplication of clinical note content hinders^144,145^ clinicians in their day-to-day process when determining the current vs. out-of-date state of patients and may introduce errors that potentially lead to safety issues.^146^ Therefore, assessing text redundancy^147^ and understanding the effect of note redundancy on developed NLP models are important, as text redundancy can have an impact on model performance.^148^

## Conclusions

Large language models are very valuable assets in the field of cardiology as LLMs are able to perform numerous NLP tasks such as speech-to-text tools to optimize patient encounters, patient-centred chatbots for question answering, and machine translation and text summarization to simplify or condense clinical notes. New opportunities to improve cardiology decision-making, streamline clinical care, and provide new and rapid insights on disease progression from free text data (*Graphical Abstract*) will be developed to enhance cardiac care. The most important aspects to ensure the safe application of LLMs in clinical practice are (i) model optimization for specific clinical tasks through fine-tuning and (ii) aligning model output with the users’ needs through reinforcement learning. To ensure the correct use of LLM-based applications in cardiology, the end-users should be aware of its limitations to ensure safe implementation of such applications in cardiology.

## Supplementary data

Supplementary data are available at *European Heart Journal* online.

## Declarations

### Disclosure of Interest

All authors declare no disclosure of interest for this contribution.

## Supplementary Material

ehad838_Supplementary_Data

## Data Availability

No data were generated or analysed for this manuscript.
