# Supplementary material for: Artificial intelligence: revolutionizing cardiology with large language models
Source: Eur Heart J. 2024 Jan 3;45(5):332–45. doi: 10.1093/eurheartj/ehad838 (PMC10834163; doi:10.1093/eurheartj/ehad838)
Supplement: ehad838_Supplementary_Data [file ehad838_supplementary_data.docx]

**Supplementary material – Glossary technical NLP/LLM terms**

| **Artificial intelligence (AI):** Computer systems designed to mimic human intelligence for specific tasks. |
| --- |
| **Machine learning (ML):** A branch of artificial intelligence in which systems use known answers to a specific task to learn to handle new data for which answers are not known. |
| **Feature:** The input to an AI-system. This input can be ‘raw’ (e.g. ECG, text, images) or derived (e.g., QRS duration, number of words). It is a measurable or characteristic property of an observed phenomenon. |
| **Token and tokenization:** The individual meaningful units of text are called tokens (may be one or more words). Tokenization is the action of finding the tokens on a given document. |
| **Embedding:** Represents the mapping of the input to a vector of continuous numbers, therewith providing the user with a meaningful low-dimensional representation of the input data. |
| **Shallow learning:** A type of machine learning architecture making use of predefined features representing the underlying raw data to train models to perform predefined tasks. Examples of shallow learners are Random Forests, Conditional Random Fields or Support Vector Machines. |
| **Deep learning:** A type of machine learning architecture making use of architectures which can process any type of underlying raw data intended to learn from this underlying data. Examples of deep learning techniques are neural networks. |
| **Neural network:** An architecture designed to learn computers to process data which is inspired by the human brain. |
| **Supervised learning:** ML training technique using labeled data and training to approximate the relation between the input data and predefined labels. |
| **Semi-supervised learning:** ML training technique using small sample of labeled data and combines this with unlabeled data to train a model. Using the unlabeled data, the shape of the larger data distribution is learned and |
| **Self-supervised learning:** ML training technique using unlabeled data to obtain useful representations to aid downstream learning tasks. |
| **Unsupervised learning:** ML training technique using unlabeled datasets to identify yet undetected patterns or clusters within the input data. |
| **Reinforcement learning:** Machine learning training method based on encouraging desired behavior and discouraging undesired behavior. |
| **Attention mechanism:** A technique to weigh the importance of specific parts of the input text for the generated model output. |
| **Pre-training:** Training a model using one dataset or for a basic task (for example predicting words in sentences) which can then be used to train another model for different more specific tasks or using task-specific data. When using such a framework, the second task-specific model does not have to be built from scratch. For LLMs, pre-training is often used with the primary objective to obtain knowledge about the general structure of written language. |
| **Fine-tuning:** The parameters and architecture from a pre-trained model are used as a starting point to optimize performance for specified tasks by adding task-specific data or layers to the overall architecture and subsequently optimizing model parameters. |
| **Prefix tuning:** A pre-trained model is optimized by adding task-specific prefix layers to the input sequence which are optimized for the down-stream task. |
| **Hard prompt tuning:** A pre-trained model is optimized by presenting the model with various input prompts requesting the same task to optimize task performance, e.g., the provided model input is changed to optimize for a specific down-stream task. |
| **Soft prompt tuning:** A pre-trained model is optimized by concatenating the input embeddings to a vector which is optimized using backpropagation to improve task performance. |
| **Prompt:** The input (i.e., message) which is used to initiate a response from a model. |
| **Prompt engineering:** Developing and optimizing prompts which can in turn be used to efficiently communicate with models. |
| **Prompt chaining:** A technique to dynamically interpret the end-users input and to generate an appropriate response. |
| **Transformers agent:** An API which analyzes the input sequence and selects |
| **Named entity recognition (NER):** The detection and categorization of important elements in free text, indicating the key subjects of a paragraph. |
| **Language model (LM):** A model representing the probability distribution of different words within a sentence. |
| **Rule-based model:** Explicit programming of rules to analyze underlying data. |
| **Large language model (LLM):** Referring to a language model based on neural networks which is trained through self-supervised or semi-supervised learning techniques. The trained neural network usually consists of millions to billions of weights. There are different types of LLMs |
| **Recurrent neural network (RRN):** Type of neural network which can represent temporal sequences. It enables the modeling of time-dependent and sequential data problems by considering the current input as well as memorize previous inputs. Types of such networks are long short-term memory (LSTM) networks or gated recurrent unit (GRU) cell networks. |
| **Sequence-to-sequence based LLM:** Class of recurrent neural network architectures often used for specific tasks like machine translation, question answering, text summarization or chatbot functions. |
| **Autoencoder based LLM:** Type of neural network which can be used to discover hidden patterns within the data by representing the input as a compressed representation using features. |
| **Transformer based LLM:** Type of neural network which can be learned the context of underlying data by assessing relationships in the sequential data. |
| **Recursive neural network-based (RvNN) LLM:** Type of neural network that to learn detailed and structured information to handle hierarchical data. This type of model is most used for sentiment analysis of written text. |
| **Hierarchical model:** Designed to handle text in different levels of granularity (documents, paragraphs, sentences) and often used for document classification. |
